# Extract from mango mistletoes *Dendrophthoe pentandra* ameliorates TNBS-induced colitis by regulating CD4+ T cells in mesenteric lymph nodes

**DOI:** 10.1186/s12906-017-1973-z

**Published:** 2017-09-25

**Authors:** Agustina Tri Endharti, Sofy Permana

**Affiliations:** 1 Department of Parasitology, Faculty of Medicine, Brawijaya University, Veteran Road, Malang, East Java 65145 Indonesia; 2 Doctoral Program in Biomedical Sciences, Faculty of Medicine, Brawijaya University, Malang, East Java Indonesia; 3 Departement of Biology, Faculty of Mathematics and Natural Sciences, Brawijaya University, Malang, East Java Indonesia

**Keywords:** Dendrophthoe pentandra, IL10, Il-17, Trinitrobenzosulfonic acid, Th17, Treg

## Abstract

**Background:**

Mango mistletoes *Dendrophthoe pentandra (*MMDP) extract has attracted interest due to its pharmacological properties, including gastro protective effects. The aim of this study was to investigate whether MMDP extract could increase Foxp3 regulatory T cells and inhibits development of Th17 cells.

**Methods:**

Colitis was induced in Balb/c mice by rectal administration of 2,4,6-trinitrobenzene sulfonic acid (TNBS). The mice were randomly divided into five groups comprising group1 receiving vehicle (the negative control), group 2–5 receiving TNBS, group 3–5 orally receiving either MMDP extract 150, 300 and 600 mg/kgBW for 7 days after TNBS administration. On day 8 of the experiment, the colon tissues were removed for histological examination, cytokine and myeloperoxidase (MPO) measurement. T-cells sub-population in mesenteric lymph nodes were analyzed by flow cytometer.

**Results:**

MMDP extract potently suppressed colon shortening and MPO in mice with TNBS-induced colitis. Administration of the extract significantly decreased the severity of TNBS-induced colitis in a dose-dependent manner. The extract significantly attenuated the loss of body weight (*p* < 0.05). These effects were associated with a remarkable amelioration of the disruption of the colonic architecture, significant reduction of the colonic MPO (*p* < 0.05). The extract lowered the levels of Th17-associated cytokines but increased the production of Treg-associated cytokines in mesenteric lymph node cells.

**Conclusion:**

Our results suggest that MMDP has the therapeutic potential to ameliorate TNBS-induced colitis symptoms revealed by histological change and inhibit IL-17 production.

## Background

*Dendrophthoe pentandra* is an Indonesian mistletoe species that belongs to the family Loranthaceae. It grows on host plants such as the mango tree. Mango mistletoe (or *benalu mangga* in Indonesian) is a semi-parasitic plant that has medicinal properties. Although mango mistletoes are considered unwanted. *D. pentandra* has been shown to possess potent anticancer activity. Additionally, in Indonesia it is used to treat hypertension, diabetes, coughs, and as a diuretic [1–3]. Phytochemical studies have demonstrated that flavonoids are the main active fraction of *D. pentandra* leaves. Previous study reported that the methanol extract of *D. pentandra* leaves showed strong antioxidant activity in various in vitro models [4, 5]. Another study described that the methanol extract of *D. pentandra* leaves exhibited a reduction in granuloma formation in mice [6]. It showed that the extract of *D. pentandra* leaves had the ability to reduce inflammation might be effective as anti-inflammatory in chronic conditions. Considering all these study we come to the fact that the anti-inflammatory activity of the methanol extract of *D. pentandra* in both acute and chronic inflammatory conditions, mostly associated with active ingredient flavonoids [7, 8].

There are limitations in both efficacy and safety of current treatment for inflammatory bowel disease (IBD). Previous studies have shown that CD4^+^ T helper Th1 and Th2 cells are essential in the pathogenesis of IBD [2, 3]. Innate and adaptive immunity of the host is thought to be involved in the pathogenesis of chronic colitis. The intra rectal administration of the ethanol solution of 2,4,6-trinitrobenzene sulfonic acid (TNBS) is used to induce intestinal inflammation in animal models that exhibit many characteristic features of IBD in humans, including severe inflammation associated with diarrhea and weight loss [9–15]. Among the immune cells involved in this process T cells and macrophages increase the secretion at the inflamed sites increase the secretion of inflammatory mediators such as IL-17, which promote and persist [16–18] In contrast, regulatory T cells (Tregs) are a specialized population of CD4^+^ T cells that act as mediators to diminish inflammatory responses and prevent autoimmunity [19–21]. Th17 is a new subtype of effector Th17cells that has been reported to play a key pathogenic role in chronic inflammatory conditions, including IBD. Roles of Th17 cells in intestinal pathology and homeostasis remain poorly understood. As described earlier, Th17 cells secrete IL-17, a pro-inflammatory cytokine that worsens chronic inflammation. Therefore, a therapeutic approach to suppress Th17 cell differentiation or to induce Treg cell differentiation can be effective in chronic colitis treatment. Previous study reported that *quercetin* contained in *D pentandra* has *gastro-*protective effect [9]. In this study, we present evidence that the ethanol extract of mango mistletoe *D. pentandra* (MMDP) has anti-inflammatory properties in vivo. The mechanisms by which the MMDP extract modulates Th17 cell differentiation in the gut are yet to be clarified. Furthermore, the effect of MMDP extract on T cells in context of the adaptive immune response has not been explored. Therefore, we investigated the effect of the MMDP extract on T cell differentiation in mesenteric lymph nodes of mice with TNBS-induced colitis.

## Methods

### Plant material and extraction

The dried plant material was mixed and macerated with absolute ethanol at a 1:20 ratio (100 g in 1 L solvent) for 7 days. Then the extract was filtrated through Whatman No 1 filter paper and then followed by rotor- evaporated the supernatant by using the BUCHI Switzerland Rotary Evaporator to remove the ethanol and to obtain concentrated, oily extract. The crude extracts were then kept in −20 °C.

MMDP leaves were collected from Probolinggo, East-Java, Indonesia and were identified and authenticated by biologists also the specimen be deposited in an official herbarium that is located at Department of Biology, Universitas Brawijaya (specimen No.0157/Taxonomy). The leaves (1.5 kg) were dried for 5 days, the dried plant material was powdered. The dried powder was subjected to extraction by maceration with 90% ethanol (1:20 ratio, 100 g in 1 L solvent) for 72 h. The maceration process was repeated three times in 24 h cycles. The resulting extraction was filtered through Whatman filter paper and then concentrated at ±60 °C under reduced pressure by using a rotary evaporator to obtain a solid form of the extract. The quercetin content from MMDP extraction was 1.15 μg/g dry weight that quantified by thin layer chromatography (TLC).

### Animals & study groups

Female Balb/c mice, aged 8–10 weeks, weight between 20 and 22 g were group-housed in cages with wire-net floors in a room maintained at 24-25 °C and a relative humidity 50–55%. Mice were given normal drinking water ad libitum and fed a standard pellet diet during the experimental period. Mice were housed 5–6 to a cage with free access to food and water on a 12 h light/12 h dark cycle. The experiments were performed in accordance with the guidelines and approval (No.160-KEP-UB) of the Institutional Animal Care and Use Committee of Brawijaya University and followed institutional requirements concerning the care and handling of animals according to Guiding Principles for the Care and Use of Animals for Scientific Purposes in the Institutional Animal Care and Use Committee (IACUC). The mice were randomly divided into five groups, each containing 10 mice. Group1 received 100 μL of 50% ethanol-phosphate-buffered saline (PBS) by rectal administration. Group 2–5 received a single rectal administration of 0.5 mg of trinitrobenzene sulfonic acid (TNBS) in 50% ethanol. MMDP extract was dissolved in 100 uL PBS and group 3–5 orally received daily the MMDP extract either at 150, 300 and 600 mg/kg body weight.

### TNBS-induced acute colitis in mice

**Fig. 1:**
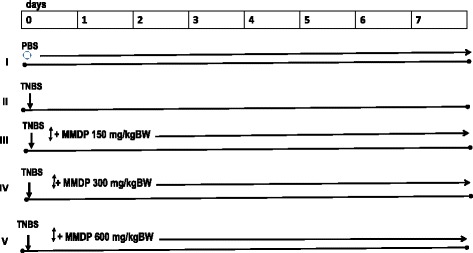
The experiment design of mice was induced Colitis and MMDP administration. The mice were randomly divided into five groups, each containing 6 mice: I group received 100 μL of 50% ethanol-phosphate buffered solution (PBS) by rectal needle; II group was given a single rectum injection of 0.5 mg of TNBS only; III-V groups were treated MMDP (150 mg/kg BW, 300 mg/kg BW and 600 mg/kg BW), respectively. The administrated of MMDP were started from the 1st until 7th days. At the end of 7 days, mice in all groups were sacrificed. ○ = PBS, ↓ = TNBS, ↕ = MMDP

Colitis was induced in mice by intra-rectal administration of 2,4,6-trinitrobenzene sulfonic acid (TNBS) by using the procedure described by Sang et al. [15]. Briefly, colitis was induced by the intra rectal administration of 0.5 mg TNBS (Sigma Chemical Co., St. Louis, MO, USA) dissolved in 50% ethanol. The volume of TNBS enema was 100 μl. To induce acute colitis, the TNBS was slowly injected into the lumen of the colon via a thin round-tip needle attached to a 1 mL syringe with mice under pentobarbital anesthesia following instillation, the animals were maintained in a head-down position for 2–3 min to prevent instillation leakage [22]. Mice were randomly divided into five groups (Fig. 1). Development of colitis was assessed daily by using an occult blood detection kit (Hemoccult). Mice experienced bloody diarrhea and a significant loss of body weight. The mice were then sacrificed at the end of 7 days. Colon tissue was removed and cleaned, then subjected to ELISA, flowcytometry and histological examination.

### Clinical assessment of colitis

Body weight, diarrhea scores, and bleeding scores were assessed daily as previously described [23]. Body weight change was monitored. The body weights and occult blood test results were recorded.

### Myeloperoxidase (MPO) activity

The activity of the enzyme MPO was used to assess the infiltration of neutrophils. Briefly, colonic proteins were extracted by lysing cells in 3 mM EDTA, 10% glycerol (pH 7.4), 200 mM NaCl, 1 mM phenyl methyl sulfonyl fluoride (PMSF), and 10 nM Tris. MPO levels were measured in the protein extracts by using a mouse MPO ELISA kit (Elabscience) according to the manufacturer’s instructions.

### Histological score of colitis

For histological examination, the colonic tissue was fixed in 10% formalin, dehydrated, paraffin-embedded, processed, sliced into 4-μm-thick sections, and stained with hematoxylin and eosin. The microscopic cross-sections of the colons were histologically investigated. Histological changes were graded semi-quantitatively from 0 to 4 according to previously described criteria as follows: 0) no signs of inflammation. 1) Very low level of leukocyte infiltration. 2) Low level of leukocyte infiltration. 3) High level of leukocyte infiltration, high vascular density and thickening of the colon wall. 4) Severe ulceration, transmural infiltration, loss of goblet cells, high vascular density, and thickening of the colon wall. All slides were evaluated using light microscopy and scored by an independent pathologist blinded to the experimental groups.

### Isolation and culture of mesenteric lymph nodes (MLNs) cells

MLN cells were isolated and transferred to ice cold sterile Hank’s balanced salt solution. The nodes were disrupted and passed through a nylon mesh (70 μm pore size). A 96-well plate was pre-coated overnight with anti-CD3 (2 μg/mL) in PBS. Single-cell suspensions of 10^6^ cells/mL were incubated in RPMI 1640 with 10% fetal calf serum and 100 IU/mL penicillin/streptomycin for 72 h in the presence of anti-CD28 antibodies (eBioscience, San Diego, CA, USA). Cell cultures were maintained in a humidified incubator at 37 °C with 5% CO_2_. Culture supernatant was collected at 72 h and then assayed for cytokines (IL-10 and IL-17) by using ELISA kits, as per the manufacturer’s instructions (R&D Systems, Minneapolis, MN, USA).

### Flow Cytometry and intracellular staining

All antibodies used for cell labeling were purchased from eBioscience. For intracellular cytokine measurement, MLN cells were stimulated for 5 h with PMA (1 μg/mL, Sigma Aldrich) and ionomycin (50 μg/mL, BD Biosciences) in the presence of monensin (0.1 mg/mL, Sigma Aldrich) and placed in a 37 °C and 5% CO_2_. MLN cells were washed with PBS and surface-labeled with anti-CD4 –FITC (Biolegend, Uithoorn, Netherlands) and anti CD25-PE (BD Biosciences). MLN cells were fixed and permeabilized (Cytofix/Cytoperm, BD Biosciences) and stained intra cellularly with anti-IL-17-PE (Biolegend, Uithoorn, Netherlands) and anti-FoxP3-PerCP (BD Biosciences). The stained cells were analyzed using FACS Calibur, and the data were analyzed using Cell Quest Pro software.

### Statistical analysis

The data were reported as mean ± standard deviation. The statistical significance was evaluated by using one-way analysis of variance (*p* < 0.05), followed by a post hoc Tukey test.

## Results

### MMDP extract ameliorates TNBS-induced acute colitis

**Fig. 2:**
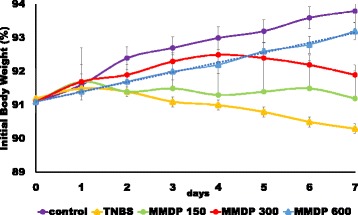
Mango Mistletoe *Dendrophthoe pentandra* (MMDP) extract protects against trinitrobenzene sulfonic acid (TNBS)-induced colitis. Mice with TNBS-induced colitis were treated with MMDP extract (150, 300, and 600 mg/kg). Change in body weight was monitored for 7 days. The changes (percentage of original body weight) have been plotted. Results represent the mean ± SD from 6 mice per group. ∗*p* < 0.05, ∗∗*p* < 0.001

Colitis was induced by the intra rectal administration of TNBS. Mice treated with TNBS developed severe bloody diarrhea accompanied by an extensive wasting disease. The MMDP extract was administered orally at different doses for 7 days after the induction of colitis. Wasting disease was ameliorated in the mice treated with the extract compared to that in the mice treated with TNBS, as assessed by the loss of body weight as well as by microscopic analysis. After administration of MMDP extract at dose dependent, a reduction in loss of body weight was observed and sustained during the 7 days (Fig. 2).

### MMDP extract attenuates TNBS induced colonic damage

**Fig. 3:**
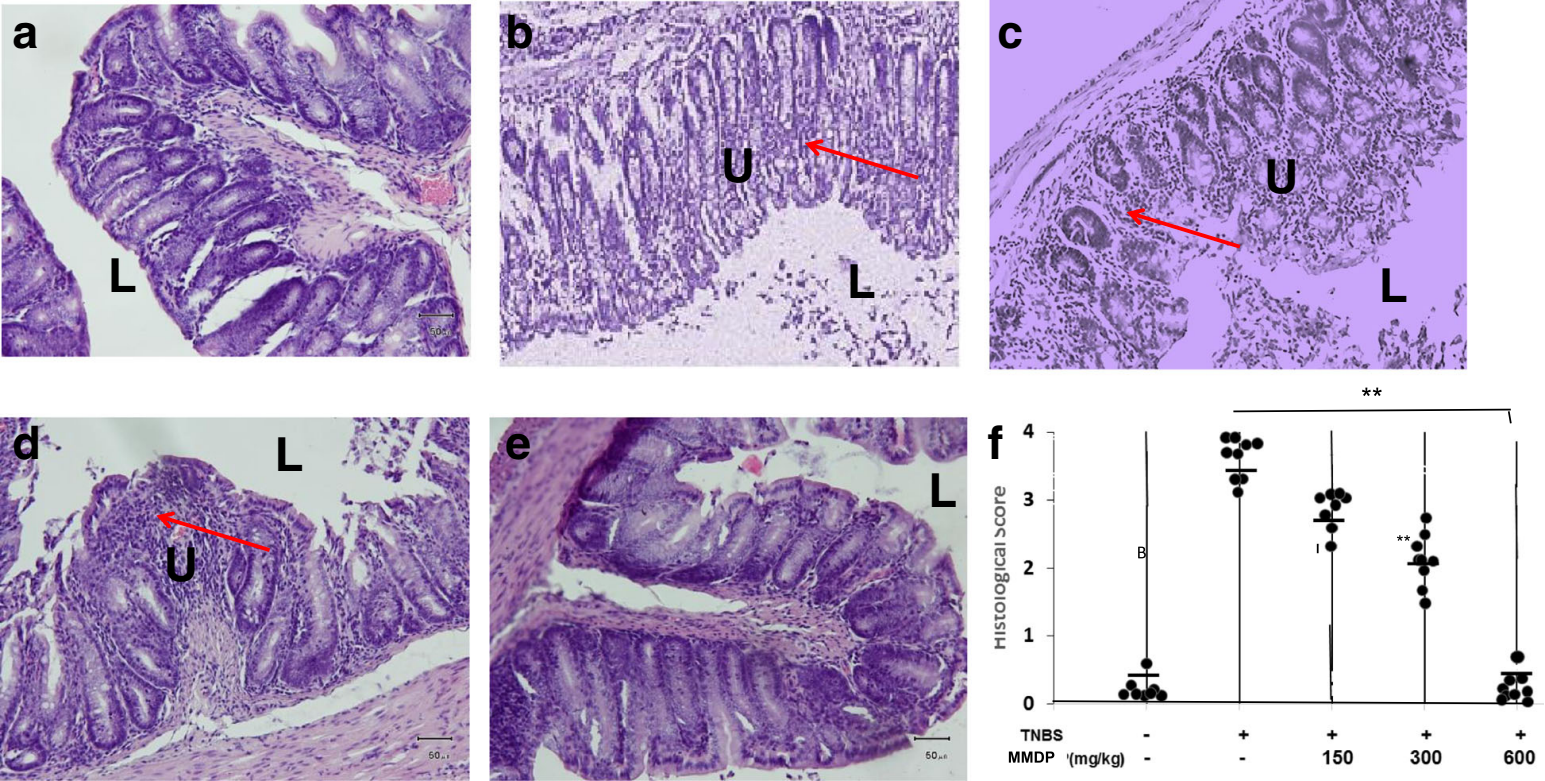
Histological changes in mice with acute trinitrobenzene sulfonic acid (TNBS) induced colitis after 7 days of treatment with mango mistletoe *Dendrophthoe pentandra* (MMDP) extract. A. Mice were sacrificed at the end of 7 days, and the colons were excised and fixed. Representative hematoxylin and eosin (H&E)-stained colonic sections of mice treated with or without the MMDP extract are shown. **a**: Mice were given untreated water as the control; **b**: Mice were given TNBS only; **c**–**e**: Mice were treated with MMDP extract (150, 300, and 600 mg/kg) respectively are shown and the averages of each group are shown as horizontal bars (total, *n* = 6). **f**. Histological scores in colonic sections from each group stained with H&E are also shown. L: Gut Lumen, U: Ulcer, Arrow: inflammatory infiltrate. The groups are significantly different (**p < 0.001, magnification 200×)

Histological examination of the colons of the mice with TNBS-induced colitis showed evidence of mucosal congestion, erosion, loss of goblet cells, thickening of the colon wall, and high level of poly-morphonuclear infiltration. The MMDP extract significantly attenuated the signs of TNBS-induced colitis (Fig. 3). Histopathologic score assessment revealed that the therapeutic effect of the extract was dose-dependent. These findings suggest that the extract affords strong protection against TNBS-induced colonic damage.

### MMDP extract decreased MPO activity

**Fig. 4:**
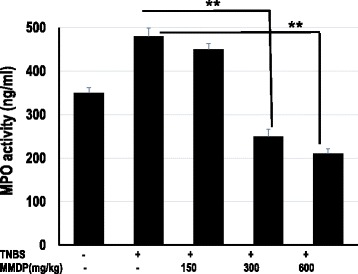
Mango mistletoe *Dendrophthoe pentandra* (MMDP) extract reduced myeloperoxidase (MPO) levels in trinitrobenzene sulfonic acid (TNBS)-induced colitis mice. The activity of the MPO was assessed to evaluate infiltration of neutrophils. MPO levels were measured in lysed colon cells. MPO activity was assessed in the colon homogenates. Results shown are mean ± SD, with *n* = 6 replicates in each group**.** **, *p* < 0.001

Colonic injury due to TNBS administration was also characterized by an increase in MPO activity, indicative of neutrophil infiltration in the inflamed tissue. The TNBS-only group showed extensive ulceration, with severe inflammatory cell infiltration. Mice from the TNBS-only group demonstrated the highest colon MPO activity, while treatment with 150 mg/kg of MMDP extract could not inhibited the MPO activity (*p* > 0.05), and mice treated with 300 and 600 mg/kg of the extract showed less MPO activity (*p* < 0.05). The MPO levels in the TNBS-only group were significantly higher than those in the MMDP extract treatment group (*p* < 0.05). MPO levels in the groups treated with 300 and 600 mg/kg extract significantly decreased compared to those in the TNBS-only group whereas treatment with the extract at 150 mg/kg did not affect the MPO levels in mice with TNBS-induced colitis (Fig. 4). The data suggest that the administration of the MMDP extract to TNBS-treated mice at 150, 300 and 600 mg/kg/day significantly prevented neutrophil infiltration, as assessed by MPO activity. Consistent with the histological changes, TNBS significantly increased colonic MPO activity. In contrast, MMDP extract-treated mice showed decreased colonic MPO activity.

### Distinct effects of MMDP extract on the presentation of Treg and Th17 cells in the TNBS-induced colitis

**Fig. 5:**
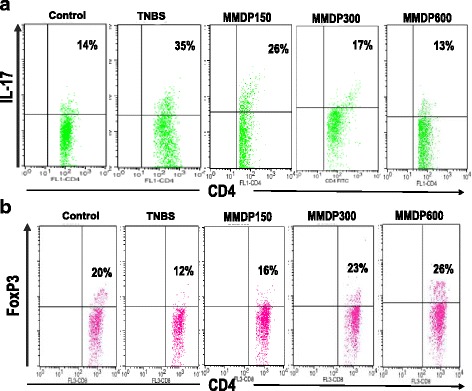
Effect of mango mistletoe *Dendrophthoe pentandra* (MMDP) extract on Foxp3 protein in CD4^+^ CD25^+^ T cells and Th17 cells. The effect of MMDP extract on the regulatory T cell (Treg) population and Th17 cells in mice with trinitrobenzene sulfonic acid (TNBS)-induced colitis is shown. **a**. Mesenteric lymph node (MLN) cells were isolated from each group and subjected to intracellular interleukin IL-17 staining. **b**. The percentage of Treg (CD4^+^ CD25^+^ Foxp3^+^) and Th17 (CD4^+^ IL-17^+^) cells were analyzed using a FACS Calibur flow cytometer (BD Biosciences). Numbers represent the percentages of IL-17 expressing CD4^+^ T cells and Foxp3-expressing CD4^+^ CD25^+^ T cells in each quadrant are shown. Percentages of cells in each quadrant are shown inside the panels. Representative results of six mice in each group are shown

Next, we assessed the effect of MMDP extract on Treg population. Foxp3 is a unique transcription factor expressed in CD4^+^ CD25^+^ T cells and has been demonstrated to be critical to Treg development. Treatment with MMDP extract suppressed TNBS-induced differentiation of Th17 cells and significantly increased the presentation of Treg cell differentiation (Fig. 5). Taken together, these results indicate that MMDP extract administration inhibits Th17 but promotes Treg responses in TNBS-induced colitis.

### MMDP extract regulates IL-10 and IL-17 production in the TNBS-induced colitis model

**Fig. 6:**
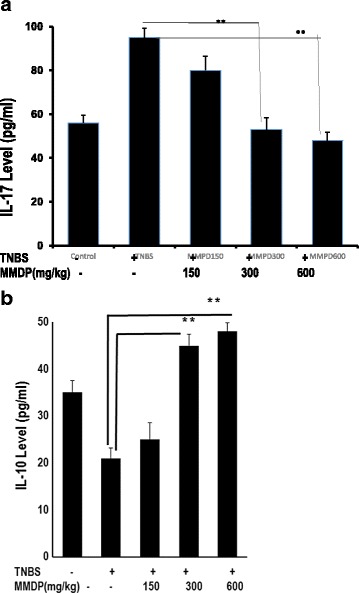
The increase in TNBS-induced IL-17 and reduction IL-10 levels in colon homogenates from the acute colitis model. **a**. ELISAs were performed for IL-17, MMDP extract suppressed TNBS-induced IL-17. **b**. ELISAs were performed for IL-10, but MMDP extract increased IL-10 production, as assessed by ELISA from supernatants of MLNs. Results shown are mean + SD, with *n* = 6 replicates in each group. ^∗^ *p* < 0.05, *p* < 0.001 versus control TNBS-induced colitis group

To determine the effect of MMDP extract on factors driving inflammatory cell responses in mice with TNBS-induced colitis, we further measured the production of cytokines critical for Th17 and Treg cell differentiation in MLNs. Oral administration of the extract increased Treg cell differentiation via enhanced Foxp3 and IL-10 expression. Furthermore, the extract also suppressed TNBS-induced IL-17 levels, but increased IL-10 production as measured by ELISA (Fig. 6). Specifically, administration of the extract at doses of 300 and 600 mg/kg caused significant decrease in IL-17 levels in TNBS-treated mice (*p* < 0.05) whereas increase in IL-10 levels (*p* < 0.05). Thus, treatment with the MMDP extract restored TNBS-induced perturbations in the differentiation of Th17 and Treg cells and the production of IL-17 and IL-10.

## Discussion

TNBS-induced colitis mice model is regarded as a classic model for the patho-immunological investigation of the colon and shares many of the histo-pathological and clinical features and pathogenesis of human IBD [22, 23]. Treatment with TNBS causes severe colitis by increasing myeloperoxidase activity [24, 25]. In this study, the effect of TNBS was reduced by MMDP extract administration, as evaluated by histology score. MMDP efficiently and dose-dependently reduced TNBS-induced colitis. It caused attenuation of weight loss, diarrhea and bleeding scores while reducing MPO activity. Our results suggest that daily MMDP extract in this murine model significantly inhibited the progression of colitis.

Colonic inflammation was assessed by evaluation of MPO activity in the colon tissue. This study suggested that the primary chemo-attractants for neutrophils are pro-inflammatory cytokines, which regulate endothelial molecule expression on vascular endothelial cells and promote neutrophil adherence to these cells [25–27]. These data suggest that oral administration of the MMDP extract significantly suppressed TNBS-induced MPO activity. Our study revealed, for the first time, an important role for MMDP extract in an immuno-modulatory effect in colitis. IL-17 accelerates chronic inflammation [28–30]. This immune response is suppressed by Treg cells, which secrete IL-10. Therefore, to control chronic inflammation, the development of regulators for T cell differentiation has been proposed.

Colonic inflammation, which is characterized by an intense neutrophil infiltration, was assessed by evaluating the MPO activity in the tissue. MMDP treatment was able to attenuate neutrophil migration and infiltration as indicated by its ability to reduce the levels of MPO. The colitis-induced body weight loss in mice over the course of our study was attenuated by MMDP treatment. Thus, preliminary findings have led us to propose that the MMDP extract has anti-inflammatory effects. It has been suggested that the main attractive sub-stances for neutrophils are pro-inflammatory cytokines [31–33].

Furthermore, our work highlights the fact that MMDP specifically interacts with the host immune system to exert its immuno-regulatory activity. It is widely accepted that TNBS-induced colitis is mediated by a dominant Th17 immune response and a deficiency of Treg responses [33–35]. In this study, we found that, in the progression of TNBS-induced colitis, treatment with MMDP extract significantly decreased the percentage of Th17 cells. Simultaneously, MMDP treatment markedly increased the percentage of CD4^+^ Foxp3^+^ (Treg cells) when compared with untreated TNBS-induced colitis group. This implies that the rehabilitating effect of MMDP in colitis is achieved by restoration of the balance between CD4^+^ T cell subsets.

The MMDP extract decreased IL-17, a Th17-associated cytokine, whereas the production of IL-10 from Treg cells in MLNs of the colonic tissue is enhanced in MMDP extract-treated mice. Our results have shown that MMDP extract was effective in protecting the colon from TNBS. We guess that Th17 was suppressed in the groups of MMDP-treated mice through its defective production. This finding confirms that treatment with MMDP extract could inhibit the differentiation of Th17 cells as indicated by the inhibition of IL-17 production. This study indicate that MMDP extract have the ability as an anti-inflammatory activity. Our results also suggest that 600 mg/kg of MMDP extract able to reduce colitis through an inflammatory process involving IL-17. Th17 cells secrete IL-17, which recruits monocytes and neutrophils and acts in synergy with other local inflammatory cytokines [35–37]. This is supported by a fact that quercetin able to diminish the inflammatory response [38].

Production of Treg-associated cytokines increased in MMDP extract-treated mice, suggesting that MMDP significantly attenuated inflammation and ameliorated the disease in TNBS-treated. The intestinal anti-inflammatory effect of MMDP extract including quercetin is associated with an inhibition in IL-17 production. Our study demonstrates the therapeutic potential of the MMDP extract a good candidate to be development in human IBD.

## Conclusion

In this study, we found that MMDP treatment significantly inhibited the population of CD4^+^IL-17^+^ cells and the IL-17 concentration in the supernatant. The decreased Th17-associated cytokines IL-17, whereas, the production of IL-10 of Treg in MLNs of colonic tissue enhanced in MMDP extract-treated mice. This finding confirms that MMDP treatment could inhibit the differentiation of Th17 cells by inhibiting IL-17 production, suggesting that MMDP extract contain quercetin play important role to inhibit inflammation.

## Abbreviations

MLN: Mesenteric Lymph Nodes
MMDP: Mango mistletoe *D. pentandra*
MPO: Myeloperoxidase
TNBS: 2,4,6-trinitrobenzene sulfonic acid
